# Venoarterial extroporeal membrane oxygenation for cardiogenic shock following acute myocardial infarction in adult Kawasaki disease: A case report

**DOI:** 10.1097/MD.0000000000048163

**Published:** 2026-03-27

**Authors:** Yan Chen, Qizhi Jin, Danqiong Wang, Yaxin Ning, Linya He, Jiahui Meng

**Affiliations:** a Jinhua Graduate Joint Training Base, Zhejiang Chinese Medical University, Jin Hua, Zhejiang, China; b Department of Cardiovascular Medicine, The Quzhou Affiliated Hospital of Wenzhou Medical University, Quzhou People’s Hospital, Quzhou, Zhejiang, China; c Department of Critical Care Medicine, The Quzhou Affiliated Hospital of Wenzhou Medical University, Quzhou People’s Hospital, Quzhou, Zhejiang, China.

**Keywords:** adult, cardiogenic shock, mucocutaneous lymph node syndrome, myocardial infarction, venoarterial extracorporeal membrane oxygenation

## Abstract

**Rationale:**

Kawasaki disease (KD) is an acute, self-limiting vasculitis that primarily affects infants and young children, and remains the leading cause of acquired heart disease in the pediatric population. With advancing disease course, adult survivors face a substantially elevated risk of developing secondary coronary artery abnormalities. When acute myocardial infarction (AMI) occurs in this population, outcomes are frequently poor due to the high prevalence of life-threatening complications, including malignant ventricular arrhythmias and cardiogenic shock.

**Patient concerns:**

Two young adult patients, aged approximately 30 years, presented with AMI complicated by cardiogenic shock, in the absence of conventional cardiovascular risk factors.

**Diagnoses:**

Coronary angiography demonstrated coronary artery aneurysms and multivessel coronary disease in both individuals. Given the lack of traditional cardiovascular risk factors, the AMI events were deemed secondary to long-standing coronary sequelae of antecedent KD.

**Interventions:**

Both patients were supported with venoarterial extracorporeal membrane oxygenation. This mechanical circulatory support successfully restored cardiac function and achieved hemodynamic stabilization during the acute critical phase of AMI and cardiogenic shock.

**Outcomes:**

Following venoarterial extracorporeal membrane oxygenation support, both patients achieved clinical recovery and were discharged home. However, during subsequent long-term follow-up, both individuals developed recurrent episodes of acute heart failure.

**Lessons:**

In patients with acute coronary syndrome attributable to KD-related coronary artery disease, prompt institution of mechanical circulatory support is critical when clinically indicated. This case further underscores that, even with successful acute-phase management, longitudinal follow-up and rigorous cardiac surveillance remain essential for early detection and prevention of progressive heart failure in this high-risk population.

## 1. Introduction

Kawasaki disease (KD) was first reported in Japan in 1967. It is also called mucocutaneous lymph node syndrome, which is an acute self-limiting vasculitis of unknown cause that mostly occurs in children under 5 years old.^[1]^ KD primarily affects small and medium-sized blood vessels and can lead to various complications, among which coronary artery lesions are the most severe complication of KD.^[2]^ 25% of untreated patients may develop coronary artery aneurysm (CAA),^[3]^ and CAA is in the middle and late stages. Thrombosis or progressive coronary artery stenosis can lead to myocardial ischemia, acute myocardial infarction (AMI), or sudden death.^[4]^ The number of adults with coronary artery disease caused by KD is gradually increasing.^[5]^ The prevalence of acute coronary syndrome (ACS) caused by KD is about 5%, but the survival rate is low and fatal ventricular arrhythmias and cardiogenic shock are prone to occur.^[6]^ Devices currently available to provide short-term circulatory support include intra-aortic balloon counter pulsation, venoarterial extracorporeal membrane oxygenation (V-A ECMO), and percutaneous left ventricular assist devices.^[7]^ Among these, V-A ECMO offers the advantage of providing comprehensive circulatory support and facilitating pulmonary gas exchange, rapidly restoring organ perfusion in cases of right heart, left heart, or biventricular failure. Thus, its use has been increasingly adopted in the management of refractory cardiogenic shock.^[8]^

## 2. Case 1

**Figure 1.**
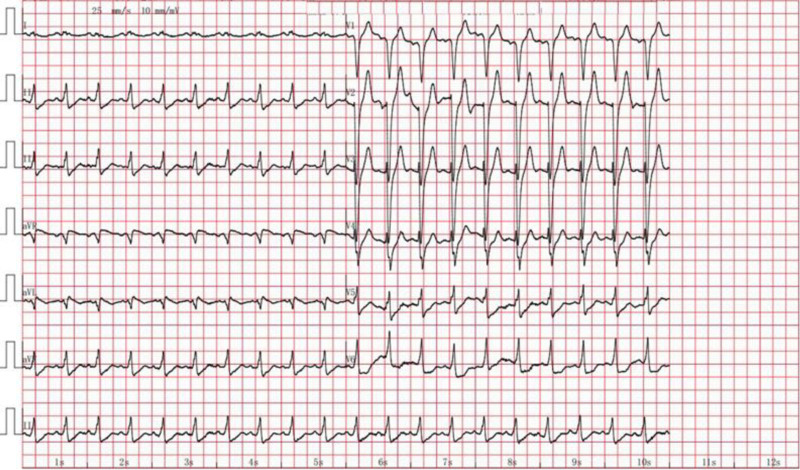
First ECG at admission: The first ECG after being transferred to our hospital: sinus tachycardia, complete left bundle branch block, and ST-segment depression of the inferior and anterolateral walls. ECG = electrocardiogram.

**Figure 2.**
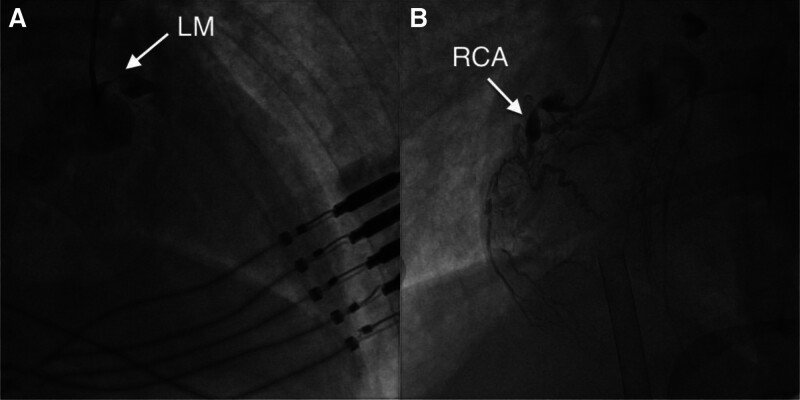
Coronary angiography: (A) The left anterior oblique view shows severe stenosis (about 90%) of the proximal right coronary artery, accompanied by an aneurysm and complete occlusion of the vessel distal to the aneurysm. (B) The head view shows an aneurysm located in the midsection of the left main trunk, which subsequently became completely occluded. LM = left main coronary artery, RCA = right coronary artery.

A male in his thirties was admitted to the hospital due to recurrent chest tightness for 1 week and followed by chest pain lasting more than 4 hours. The patient experienced recurrent chest tightness during brisk walking 1 week prior to admission, without seeking medical attention or taking any medication. More than 4 hours ago, he suddenly developed retrosternal stabbing pain, followed by syncope. After regaining consciousness, he continued to experience persistent chest tightness and chest pain. Initial troponin I level was 0.02 ng/mL (normal range: < 0.04 ng/mL). The electrocardiogram (ECG) revealed a complete left bundle branch block, ST-segment depression in the inferior wall and leads V4-V6, and T wave was low and flat. Chest computed tomography demonstrated bilateral pulmonary edema and pleural effusion. The clinical picture was suggestive of acute non-ST-segment elevation myocardial infarction (STEMI). The patient received 300 mg aspirin, 300 mg clopidogrel, and 40 mg atorvastatin orally before transfer to our hospital. The review of the ECG (Fig. 1) revealed a complete left bundle branch block and ST-segment depression in the inferior and anterolateral walls. Emergency coronary angiography (Fig. 2) revealed aneurysmal expansion in the middle segment of the left main artery (LMA), complete occlusion of the post-aneurysmal blood vessels, and chronic occlusion of the proximal segment of the right coronary artery (RCA). Percutaneous transluminal coronary angioplasty was performed on the LMA. The patient experienced cardiac arrest during the procedure, prompting the initiation of V-A ECMO support. After the surgery, the patient was transferred to the intensive care unit (ICU). He has no history of illness and tobacco or alcohol addiction.

Upon admission to the ICU, the patient was comatose, with a temperature of 35.8°C, pulse rate of 91 beats/minute, respiratory rate of 12 beats/minute, and blood pressure of 81/77 mm Hg, maintained with norepinephrine at 0.75 µg/kg/minute and epinephrine at 0.25 µg/kg/minute. The patient was intubated and placed on mechanical ventilation in PC/AC mode (PC = 15 cm H_2_O, PEEP 10 cm H_2_O). V-A ECMO was operational, with a flow rate of 3.55 L/minute and a rotation speed of 2495 r/minute. Physical examination revealed coarse breath sounds and distinct moist rales in both lungs, diminished heart sounds, while the remainder of the physical examination was unremarkable.

Ancillary tests revealed the following results: complete blood count indicated a white blood cell count of 14.8 × 10^9^/L, a neutrophil percentage of 81.8%, a platelet count of 267 × 10^9^/L, and a hemoglobin level of 132 g/L; serum biochemistry showed an alanine aminotransferase level of 477.8 U/L and a creatinine level of 126.3 μmol/L; cardiac enzyme profile demonstrated a troponin I level of 0.233 μg/L, plasma brain natriuretic peptide of 578.40 pg/ml, creatine kinase of 3237.5 U/L, creatine kinase isoenzyme of 679.1 U/L, and lactate dehydrogenase of 1561.3 U/L; right radial artery blood gas analysis indicated a potential of hydrogen of 7.217, arterial blood carbon dioxide partial pressure of 31.5 mm Hg, bicarbonate- of 12.3 mmol/L, blood lactate of 19 mmol/L, and a oxygenation index ratio of 340 mm Hg; cardiac ultrasound revealed left heart enlargement, diffuse left ventricular wall hypokinesia, and significantly reduced left ventricular systolic function, left ventricular ejection fraction (LVEF) = 8%.

The diagnoses for admission to ICU include acute inferior wall myocardial infarction, acute lateral wall myocardial infarction, Killip IV, cardiogenic shock, cardiac arrest, post-cardiopulmonary resuscitation, acute respiratory failure, KD, pleural effusion, hepatic insufficiency, acute renal insufficiency, and metabolic acidosis.

**Figure 3.**
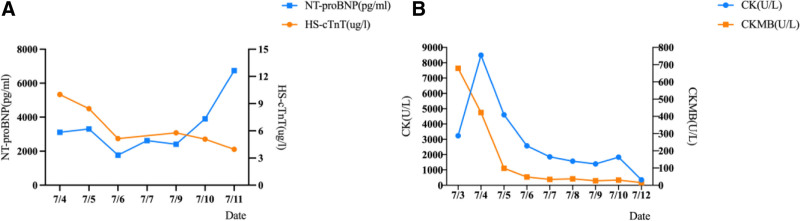
Myocardial enzyme trends: (A) Changes in N-terminal pro-B-type natriuretic peptide (NT-proBNP) and high-sensitivity troponin T (HS-cTnT) within 8 days after the patient’s admission to the ICU. (B) Changes in creatine kinase and creatine kinase isoenzyme within 10 days after the patient’s admission to the ICU. CK = creatine kinase, CKMB = creatine kinase isoenzyme, ICU = intensive care unit.

Following admission to the ICU, the patient received several interventions, including mechanical ventilation, V-A ECMO support, continuous renal replacement therapy, prone positioning ventilation, epinephrine, and norepinephrine to maintain blood pressure. Hemodynamic parameters, cardiac echocardiography, and myocardial enzyme spectrum were monitored dynamically throughout the process (Fig. 3). The treatment parameters of V-A ECMO, dosages of vasoactive drugs, and monitoring indicators are presented in Table 1. On the second day of V-A ECMO treatment, the patient’s LVEF improved to 19%. During this period, the patient experienced recurrent atrial fibrillation. By the seventh day, the patient’s circulation stabilized, and a repeat LVEF measurement indicated recovery to 32%, accompanied by a significant decrease in lactate levels. Blood pressure remained stable with the use of low-dose vasoactive drugs, leading to the decision to withdraw V-A ECMO. Treatment continued with diuretics, digoxin, aspirin, and other medications. Due to the development of a pulmonary infection, antibiotic therapy was administered. On the day 33, the patient’s creatine kinase isoenzyme levels were elevated, and a repeat ECG indicated ST-segment depression. A repeat cranial computed tomography scan revealed cerebral hemorrhage following cerebral infarction, prompting treatment with clopidogrel 75 mg once daily after a comprehensive evaluation by the cardiology and neurosurgery departments. The patient was transferred back to the local hospital for continued treatment. Half a month after discharge, the patient was readmitted to the ICU due to cardiac arrest and cardiogenic shock. After receiving ventilatory support from a respirator and pharmacological treatment, the patient’s condition improved, and he was ultimately discharged. During follow-up, the patient underwent coronary artery bypass grafting at another hospital and has been on long-term oral treatment with aspirin, clopidogrel. And fortunately, the patient’s condition remaining stable.

**Table 1:** Main parameters and monitoring indicators of venoarterial extracorporeal membrane oxygenation treatment.

Treatment Time	Blood Flow (L/min)	Gas Flow (L/min)	Oxygen concentration (%)	Heparin (mg/h)	Vasoactive Agents (µg/kg/min)		MAP (mm Hg)	SpO_2_ (%)	Hb (g/L)	pH	PaO_2_/FiO_2_% (mm Hg)	PaCO_2_ (mm Hg)	HCO_3_^-^ (mmol/L)	Lac (mmol/L)	ACT (s)
					Norepinephrine	Epinephrine									
Day 1	3.54	4.00	100	-	0.796	0.796	65	-	140	7.217	340	31.5	12.3	19	266
Day 2	3.53	2.90	40	6	0.955	0.239	78	100	111	7.397	620	32.9	19.8	3.2	212
Day 3	3.12	2.14	40	2	0.279	0.040	88	100	83	7.479	632	33.3	24.5	1.6	200
Day 4	2.70	1.54	40	3	0.080	0.020	81	100	86	7.572	635	28.4	26.2	1.4	194
Day 5	2.25	1.50	50	3	0.020	0.020	75	100	80	7.449	446	33.1	22.6	1.2	182
Day 6	2.01	1.55	35	5	0.020	0.020	98	100	94	7.441	320	36.3	24.3	1.4	190
Day 7	1.53	1.50	35	6	0.020	0.020	80	100	75	7.412	326	26.7	16.7	1.4	185

1 mm Hg ≈ 0.133 k Pa. Vasoactive agent concentrations were calculated based on standard body weight. - indicates not measured.

ACT = activation clotting time, Hb = hemoglobin, HCO_3_^-^ = bicarbonate, Lac = blood lactate, MAP = mean arterial pressure, PaCO_2_ = arterial blood carbon dioxide partial pressure, PaO_2_/FiO_2_% = oxygenation index, SpO_2_% = blood oxygen saturation.

## 3. Case 2

**Figure 4.**
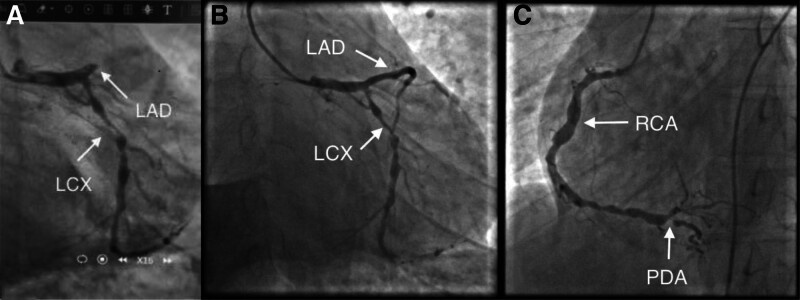
Coronary angiography: (A) Coronary angiography performed at another hospital showed occlusion in the proximal segment of the left anterior descending artery (LAD), along with diffuse lesions in the left circumflex artery (LCX) and 90% stenosis at the most severe site. (B) Coronary angiography performed at our hospital showed that following stent implantation in the LAD, the stent in the proximal to midsection was incompletely expanded, and the distal segment has diffuse lesions, with the most severe stenosis measuring 80%. Additionally, the left circumflex artery (LCX) displayed diffuse disease, with the most severe stenosis also at 90%. (C) Coronary angiography at our hospital, from the left anterior oblique view, showed diffuse lesions in the right coronary artery (RCA) accompanied by multiple aneurysms and severe stenosis (about 90%) of the posterior descending artery. LAD = left anterior descending artery, LCX = left circumflex artery, PDA = posterior descending artery, RCA = right coronary artery.

A male patient in his thirties was admitted to the hospital after experiencing chest pain for 18 hours, with a recurrence occurring 9 hours prior to admission. The patient experienced sudden chest pain 18 hours before admission, which resolved spontaneously. However, the chest pain recurred and persisted for 9 hours before admission, accompanied by chest tightness. An ECG from the outside hospital indicated high peak T waves, abnormal Q waves in the lateral wall, and mild ST-T changes; troponin T levels were normal, emergency percutaneous coronary intervention (PCI) was performed, and angiography (Fig. 4A) revealed proximal occlusion of the left anterior descending artery (LAD), diffuse lesions in the left circumflex artery (LCX) and RCA, and near-total occlusion of the posterior descending artery. Under the guidance of intravascular ultrasound, 2 stents were implanted in the LAD. During the surgery, the patient occurred ventricular fibrillation and cardiogenic shock, and V-A ECMO extracorporeal circulation support was immediately started. And then he was transferred to the ICU of our hospital after surgery. The patient had no significant medical history but was a smoker for over 10 years.

Physical examination upon admission to ICU: sedated state, temperature 36.3°C, pulse of 141 beats/minute, respiratory rate of 27 beats/minute and blood pressure of 136/111 mm Hg (maintained with norepinephrine at 0.22 µg/kg/minute). The patient was intubated (PC/AC mode, PC of 15 cm H_2_O and PEEP of 10 cm H_2_O, FiO_2_ 100%). ECMO was operational with blood flow of 2.0 L/minute and pump speed of 6100 r/minute. The patient exhibited slow light reflection, rough breathing sounds, wet sounds in both, irregular arrhythmia, and weakened heart sounds, other physical examination was normal.

**Figure 5.**
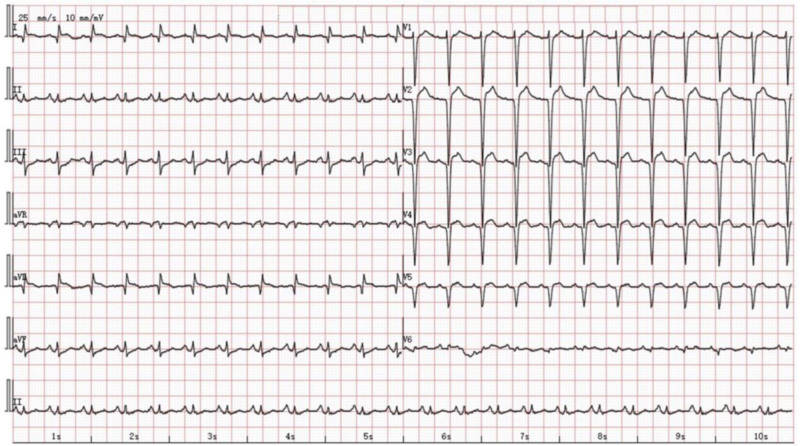
ECG on ICU admission: ECG on admission to ICU shows sinus tachycardia, the presence of abnormal Q waves in the anterior and lateral walls, arched ST-segment elevation, and T wave inversion in the high lateral wall. ECG = electrocardiogram, ICU = intensive care unit.

Auxiliary examinations revealed the following results: complete blood count indicated a white blood cell count of 31.6 × 10^9^/L, a neutrophil percentage of 92.3%, a platelet count of 321 × 10^9^/L, and a hemoglobin level of 160 g/L; serum biochemistry showed alanine aminotransferase at 140.8 U/L and creatinine at 53.6 μmol/L; cardiac enzyme profile demonstrated high-sensitivity troponin T at 8.57 μg/L, N-terminal pro-B-type natriuretic peptide at 171.8 pg/ml, creatine kinase at 7946.5 U/L, creatine kinase isoenzyme at 1346.6 U/L, and lactate dehydrogenase at 1161.1 U/L; right radial artery blood gas analysis revealed a potential of hydrogen of 7.312, arterial blood carbon dioxide partial pressure of 32.9 mm Hg, bicarbonate^-^ of 16.6 mmol/L, blood lactate of 4.3 mmol/L, and a oxygenation index ratio of 213 mm Hg; cardiac ultrasound indicated diffuse wall motion hypokinesia and reduced left ventricular systolic function (LVEF = 30%); ECG (Fig. 5) displayed sinus tachycardia, abnormal Q waves with upward convex ST-segment elevation in the anterior and lateral walls, and inverted T waves in the high lateral wall.

The diagnosis for admission to ICU includes acute anterior wall myocardial infarction, acute lateral wall myocardial infarction, Killip IV, cardiogenic shock, ventricular fibrillation, respiratory failure, metabolic acidosis, and hepatic insufficiency.

**Figure 6.**
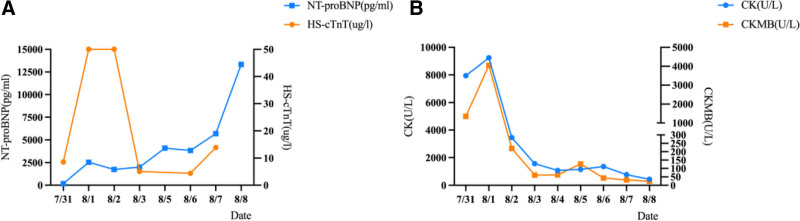
Myocardial enzyme trends: (A) Changes in N-terminal pro-B-type natriuretic peptide (NT-proBNP) and high-sensitivity troponin T (HS-cTnT) within 9 days after the patient’s admission to the ICU. (B) Changes in creatine kinase and creatine kinase isoenzyme within 9 days after the patient’s admission to the ICU. CK = creatine kinase, CKMB = creatine kinase isoenzyme, ICU = intensive care unit.

Following admission to the ICU, the patient received several interventions, including mechanical ventilation, V-A ECMO support, continuous renal replacement therapy, norepinephrine to maintain blood pressure, and aspirin, clopidogrel to antiplatelet aggregation treatments. Hemodynamic parameters, cardiac echocardiography and myocardial enzyme spectrum were monitored dynamically throughout the process (Fig. 6). The parameters for ECMO treatment, dosages of vasoactive drugs, and monitoring indicators are detailed in Table 2. On the third day, the echocardiogram showed an improvement in cardiac function, with an LVEF of 43% and the creatine kinase isoenzyme exhibited a downward trend, so we withdrew the V-A ECMO. After the patient’s condition stabilized, they were transferred to our hospital’s Department of Cardiology for a second PCI. The coronary angiography (Figs. 4B and 4C) showed that there was a stenosis of about 30% in the proximal segment of the LMA, and the dilation of stent at the proximal to middle segment of the LAD was incomplete. And there were diffuse lesions and aneurysm formation in the LAD, LCX, and RCA, with the most severe stenosis of nearly 90%. Balloon dilation was performed on the LAD, LMA, and LCX, and implanted a drug stent in the LCX. Based on the characteristics of the patient’s coronary artery lesions, the patient was diagnosed with KD. After improvement and discharge, the patient was repeatedly hospitalized for acute heart failure within 6 months but improved after treatment. The patient’s condition is currently stable, with no recurrence of acute heart failure observed to date following long-term oral treatment with aspirin, clopidogrel, and diuretics.

**Table 2:** Main parameters and monitoring indicators of venoarterial extracorporeal membrane oxygenation treatment.

Treatment Time	Blood Flow (L/min)	Gas Flow (L/min)	Oxygen concentration (%)	Heparin (mg/h)	Norepinephrine (µg/kg/min)	MAP (mm Hg)	SpO_2_ (%)	Hb (g/L)	pH	PaO_2_/FiO_2_% (mm Hg)	PaCO_2_ (mm Hg)	HCO_3_^-^ (mmol/L)	Lac (mmol/L)
Day 1	2.80	2.11	50	2	0.444	88	97	139	7.388	178	35.3	21.3	3.2
Day 2	2.81	1.67	40	4	0.133	79	99	117	7.415	308	40.1	25.7	1.5
Day 3	2.35	1.50	40	6	0.222	75	99	118	7.347	320	52.4	28.7	1.1

1 mm Hg ≈ 0.133 kPa. Norepinephrine concentrations were calculated based on standard body weight.

ACT = activation clotting time, Hb = hemoglobin, HCO_3_^-^ = bicarbonate, Lac = blood lactate, MAP = mean arterial pressure, PaCO_2_ = arterial blood carbon dioxide partial pressure, PaO_2_/FiO_2_% = oxygenation index, SpO_2_% = blood oxygen saturation.

## 4. Discussion

Early diagnosis of KD mainly relies on typical clinical manifestations. KD can be diagnosed when a fever persists for ≥ 5 days alongside at least 4 of the following main clinical features: bilateral bulbar conjunctival congestion, polymorphic rash, changes in the extremities (including redness and hard edema during the acute phase, and membranous peeling during the recovery phase), alterations in the lips and oral cavity (such as redness, dryness, cracking, peeling, or a strawberry tongue), and cervical lymph node enlargement with a diameter of ≥ 1.5 cm.^[3]^ However, diagnosis is more difficult when the clinical manifestations are atypical or incomplete, and the risk of CAA increases due to delays in diagnosis and treatments.^[9]^ Therefore, in the presence of fever and lack of 4 major clinical features, if echocardiography and coronary angiography suggest the presence of coronary artery disease, KD can also be diagnosed.^[3]^ KD damages coronary arteries aneurysms, calcifications or stenosis,^[10]^ and KD patients with large CAA often suffer from serious adverse cardiac events such as unstable angina and myocardial infarction,^[11]^ and KD-related CAA tends to occur at the proximal of LAD and RCA, and bifurcation of the LCA. As CAA increases, the coronary arteries dilate and extend from proximal to distal.^[12]^ Calcification of proximal coronary arteries is a characteristic manifestation of late-stage coronary artery disease caused by KD.^[13]^ KD-related ACS manifests as chronic total occlusion and multivessel disease.^[14]^

Patients with a history of KD may be at risk for premature atherosclerosis.^[15]^ Anzai et al found that compared with non-KD-complicated AMI, patients with KD complicated by AMI were younger and more likely to develop disease around the age of 35. At the same time, the incidence of coronary artery disease risk factors such as hypertension, diabetes, and dyslipidemia is lower and has a higher incidence of cardiac arrest and severe cardiac dysfunction.^[14]^ In adults, the prevalence of ACS caused by KD is about 5%, and the survival rate is very low. Preserving LVEF is an important factor in improving its survival rate.^[6]^ Patients with reduced LVEF are at high risk for fatal ventricular arrhythmias and cardiogenic shock.^[16,17]^ Patients with pulmonary hypertension often have refractory heart failure, so patients often need to be hospitalized repeatedly.^[18]^ V-A ECMO can improve myocardial reperfusion and reduce cardiac preload and myocardial oxygen demand, thereby preserving left ventricular function after AMI.^[17,19]^ The retrospective study results of Tsao et al showed that the impact of refractory ventricular tachycardia and ventricular fibrillation on patient survival during V-A ECMO assistance was greatly reduced, and V-A ECMO-assisted PCI treatment could improve 30-day and 1-year survival rates in patients with AMI complicated by severe cardiogenic shock.^[20]^ At the same time, Chung et al study found that the long-term survival rate of STEMI patients with severe cardiogenic shock who received primary PCI with ECMO support could reach 32.3%.^[21]^ Therefore, ECMO-assisted PCI may be meaningful to improve the survival rate of patients with STEMI complicated by severe cardiogenic shock.

## 5. Conclusion

It is essential to consider late complications of KD in young ACS patients without standard risk factors. To improve outcomes, aggressive use of extracorporeal circulation support at the onset of cardiogenic shock is critical to preserve left ventricular function. Furthermore, patients presenting with low LVEF require vigilant long-term management due to their elevated risk of refractory heart failure and repeat hospitalization.

## Acknowledgments

The authors thank the medical team for their dedicated care and collaboration.

## Author contributions

**Data curation:** Yan Chen, Yaxin Ning, Linya He, Jiahui Meng.

**Investigation:** Yan Chen.

**Methodology:** Yan Chen.

**Software:** Yan Chen.

**Supervision:** Qizhi Jin, Danqiong Wang.

**Visualization:** Yan Chen.

**Writing – original draft:** Yan Chen, Yaxin Ning, Linya He, Jiahui Meng.

**Writing – review & editing:** Yan Chen, Qizhi Jin, Danqiong Wang.
